# A unified view of spatio-temporal control of mitotic entry: Polo kinase as the key

**DOI:** 10.1098/rsob.180114

**Published:** 2018-08-22

**Authors:** Lionel Pintard, Vincent Archambault

**Affiliations:** 1 Cell Cycle and Development Team, Institut Jacques Monod, UMR7592 CNRS—Université Paris Diderot, Sorbonne Paris Cité, Ligue contre le Cancer, Paris, France; 2 Equipe labellisée, Ligue contre le Cancer, Paris, France; 3 Institut de recherche en immunologie et en cancérologie, Université de Montréal, Montréal, Québec, Canada

**Keywords:** cell cycle, mitosis, Polo, Plk1

## Abstract

The Polo kinase is an essential regulator of cell division. Its ability to regulate multiple events at distinct subcellular locations and times during mitosis is remarkable. In the last few years, a much clearer mechanistic understanding of the functions and regulation of Polo in cell division has emerged. In this regard, the importance of coupling changes in activity with changes in localization is striking, both for Polo itself and for its upstream regulators. This review brings together several new pieces of the puzzle that are gradually revealing how Polo is regulated, in space and time, to enable its functions in the early stages of mitosis in animal cells. As a result, a unified view of how mitotic entry is spatio-temporally regulated is emerging.

## 1. Polo is a multi-faceted kinase

Discovered in *Drosophila*, Polo gave its name to the Polo-like kinase (PLK) family, five of which exist in humans (although Plk5 is kinase-inactive) [1,2]. *Drosophila* Polo, mammalian Plk1, *Xenopus* Plx1 and *Caenorhabditis elegans* PLK-1 are clear orthologues at the sequence and functional levels (here, we refer to them collectively as Polo). These Ser/Thr kinases are defined by the presence of an N-terminal kinase domain (KD) and an additional C-terminal domain, termed the Polo-box domain (PBD), which engages in protein interactions [3,4]. In all these systems, Polo promotes centrosome maturation, centromere/kinetochore (KT) function, chromosome condensation, spindle assembly and function and cytokinesis [5,6]. During development, Polo also coordinates cell-cycle progression with cell polarity and cell fate determination [7–9].

**Figure 1.**
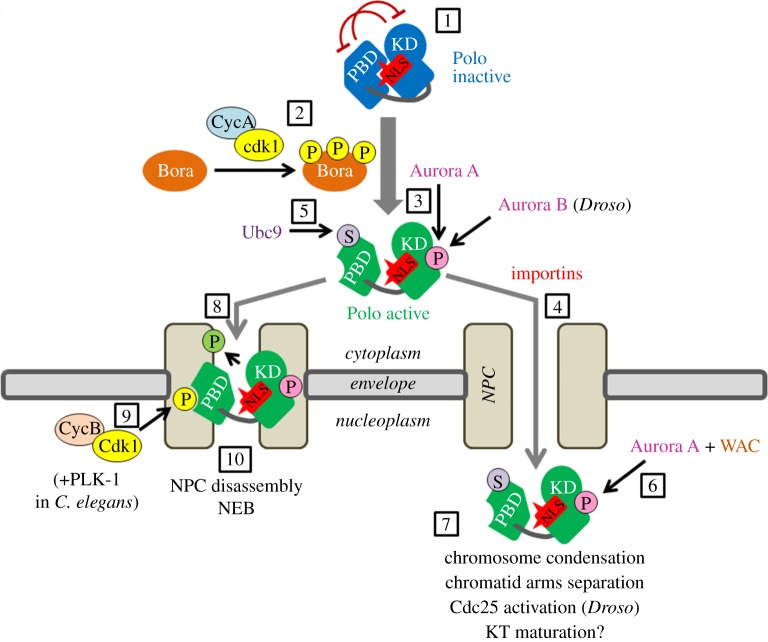
Spatio-temporal coordination of Polo kinase during mitotic entry in metazoans. 1, Polo kinase (Plk1 in humans) is kept inactive (blue) by an intramolecular interaction between its KD and its PBD, which inhibit each other (red arrows). In this conformation, the nuclear localization signal (NLS, red) of the KD of Polo is buried by the PBD, and amino acid residues of the NLS mediate the inhibitory interaction with the PBD. 2, During G2, Cyclin A–Cdk1 phosphorylates Bora at multiple sites. 3, This event facilitates the activating phosphorylation of Polo by Aurora A (Polo is phosphorylated by Aurora B independently from Bora in *Drosophila (Droso*)). 4, This phosphorylation of Polo leads to the exposure of the NLS, which becomes accessible to importins that transport active Polo (green) into the nucleoplasm. 5, In human cells, nuclear transport of Plk1 also requires SUMOylation of the PBD by Ubc9, although how this event relates to the phosphorylation, the interdomain interaction or the exposure of the NLS of Plk1 is unknown. 6, In human cells, WAC promotes sustained phosphorylation of Plk1 by Aurora A in the nucleus. 7, Once in the nucleus, active Polo promotes mitotic entry in several ways, as indicated. 8, Coincident with its activation, Polo also re-localizes to nuclear pore complexes (NPCs). 9, This targeting requires the priming phosphorylation of nucleoporins at CDK sites, presumably by Cyclin B–Cdk1, creating PBD docking sites. In *C. elegans*, PLK-1 also contributes to phosphopriming. 10, At the NPCs, Polo phosphorylates nucleoporins to induce NPC disassembly and nuclear envelope breakdown (NEB).

To regulate these different functions, Polo must be activated and dynamically recruited to distinct subcellular structures, in space and time [10]. The localization of Polo is mediated by protein interactions with the PBD. This targeting facilitates the phosphorylation of various effectors by the KD of Polo [11]. The PBD is a phospho-peptide binding domain and its interactions are thus often enhanced by prior phosphorylation of the target [12]. This priming phosphorylation is generally mediated by Cdk1 in mitosis; however, in G2 or during mitotic exit, when Cdk1 is less active, Polo tends to phosphorylate its targets to prime its subsequent binding to them (for self-priming) [4]. In addition, the PBD and the KD can engage in a reciprocal inhibitory interaction (figure 1, point 1) [13,14]. The kinase activity, PBD interactions, interdomain inhibition and localization of Polo are all subjected to regulation by post-translational modifications and structural changes [3]. Because these mechanisms work together to control Polo, they need to be understood at the molecular level to fully appreciate the place Polo occupies on the central stage of cell division. Here, we focus on recent progress made in understanding the complex spatio-temporal regulation and functions of Polo in early mitosis.

## 2. Polo function in mitotic entry revisited: Plk1 is essential for mitotic entry in human cells

The Polo kinase has long been known to promote mitotic entry in various systems. In *Xenopus* extracts, Plx1 (Polo) was isolated as a kinase required to phosphorylate and to activate Cdc25C, the phosphatase required for activation of Cyclin B–Cdk1 [15,16]. As Cyclin B–Cdk1 is the central trigger of mitotic entry, defined by nuclear envelope breakdown (NEB) and chromosome condensation, these results implied that Polo should be required for mitotic entry, acting upstream of Cyclin B–Cdk1 [17]. However, the phenotypes of the *polo* mutants isolated in *Drosophila* suggested that Polo was needed for cells to assemble functional mitotic centrosomes and a bipolar spindle, but mutant neuroblasts did enter mitosis [1]. These phenotypes could have been due to incomplete inactivation of Polo in these tissues, where maternally contributed Polo is gradually depleted in the developing fly. Consistent with a role for Polo in mitotic entry, inactivation of Plk1 (Polo in human cells) by antibody injection in non-transformed human cells caused a majority of cells to arrest in the G2 phase of the cell cycle [18]. However, subsequent inactivation of Plk1 by RNAi or with ATP-competitive chemical inhibitors induced a prometaphase arrest after a delay in G2 or prophase, suggesting that Plk1 was not required for mitotic entry in human cells progressing through normal cell cycles [19–22]. In any case, Plk1 activity was found to be essential for re-entry into mitosis after recovery from DNA damage [23]. Thus, the importance of Polo activity for mitotic entry during an unperturbed cell cycle remained questionable.

Recently, two studies revisited the question and demonstrated that complete inhibition of Plk1 can prevent mitotic entry for hours in a vast majority of hTERT-RPE1, HeLa or RKO cells, even without prior activation of the DNA damage checkpoint [24,25]. Interestingly, mitotic entry was blocked more efficiently in untransformed RPE-1 cells than in the transformed HeLa cells, probably because Plk1 is expressed at higher levels in HeLa cells, as in many cancer cell lines [24]. Overall, the different results obtained in various contexts by different groups over the years can be largely reconciled by the fact that a much lower Polo activity is sufficient to promote mitotic entry than the level of activity required for the cell to build a mitotic spindle and progress through mitosis [26]. Yet, unlike Cdk1 activity which is completely required for mitotic entry from yeast to humans [17], Polo activity may not be universally required for this transition. For example, in budding yeast, inactivation of Cdc5 (Polo) kinase results in a late anaphase arrest [27,28]. Nevertheless, the recent studies have now firmly established that Plk1 activity is required for mitotic entry in some of the most commonly used human cell lines to study mitosis [24,25].

## 3. Mechanisms of spatio-temporal regulation of Polo at the G2/M transition

The importance of Polo activity for mitotic entry is reflected by its activation and relocalization during this transition. During mitotic entry, Polo kinase is activated by phosphorylation of an evolutionarily conserved residue located in the activation loop (Thr210 in human Plk1) [29,30]. Phosphorylation at this residue is primarily mediated by Aurora A in vertebrates (figure 1, point 3) [31,32] and possibly also in *C. elegans* [33,34], and by Aurora B in *Drosophila* [35]. Aurora A is enriched at spindle poles, and phosphorylated Plk1 is first detected at centrosomes as human cells enter mitosis [10]. However, concomitant with its activation, Polo is suddenly imported to the nucleus in prophase. This relocalization of Polo has been observed in human cells, *C. elegans* embryos and *Drosophila* cells in culture [36–38]. Moreover, recent work indicates that active Polo kinase needs to enter the nucleus in order to promote mitotic entry. In an elegant set of experiments, Bruinsma *et al.* [39] showed that restricting Plk1 to the cytoplasm only (where it is activated) or to the nucleus only does not allow Plk1 to promote mitotic entry. These observations suggested the existence of a mechanism coupling Polo activation to its nuclear import.

The elucidation of the first crystal structure showing how the KD and PBD of zebrafish Plk1 interact in the inactive state helped solve this mechanism [40]. It revealed how the PBD binds to the KD in a rigid conformation incapable of catalysis, effectively inhibiting it. By examining the structure, Kachaner *et al.* [38] noticed that residues of a known bipartite nuclear localization signal (NLS) in the KD of Plk1 [41] make direct contacts with the PBD and are required for the interdomain inhibitory interaction within Polo. In this closed conformation, the NLS is masked by the PBD, presumably inaccessible to importins. Previous work had shown that phosphorylation of the activation loop in the KD abrogates the interdomain interaction [14], thus potentially exposing the NLS. Working in *Drosophila*, Kachaner *et al.* showed that phosphorylation of Polo is required for the exposure of the NLS and nuclear import of Polo (figure 1, points 3 and 4). As a result, activated Polo is rapidly sent to the nucleus [38]. As this NLS of Polo is widely conserved among eukaryotes [41], this mechanism is probably a general feature of Polo regulation.

Other mechanisms contribute to regulate Polo activation and localization during mitotic entry. In *Drosophila*, the dissociation of the PBD from the KD induced by the phosphorylation of Polo also weakens an interaction of the PBD with Map205 [38]. This protein binds the PBD of Polo and promotes the PBD–KD intramolecular interaction, thereby acting as an allosteric inhibitor of the KD [38,40]. As Map205 is a microtubule-associated protein, phosphorylation of Polo by Aurora B not only relieves Polo from its intramolecular interaction, it also promotes the release of Polo from a sequestration on microtubules, facilitating its relocalization [42,43]. This mechanism also regulates the essential role of *Drosophila* Polo in cytokinesis; however, as no Map205 orthologue has been identified in humans, it remains to be seen if other proteins similarly regulate Plk1 [42].

In human cells, the nuclear localization of Plk1 has recently been shown to depend also on the SUMOylation of the PBD at Lys492 (figure 1, point 5) [44]. This event is catalysed by Ubc9, and is facilitated by the priming phosphorylation of Ubc9 at a CDK site, which enhances its binding to Plk1. It is tempting to speculate that, like phosphorylation of the activation loop, SUMOylation induces a structural change that exposes the NLS in the KD to promote nuclear import of Plk1. The functional relationship between activating phosphorylation of the KD and SUMOylation of the PBD in triggering the nuclear import of Polo remains to be explored. Interestingly, Lys492 is also the site of mono-ubiquitination of Plk1. This event promotes the relocalization of Plk1 from KTs to the central spindle at the metaphase/anaphase transition [45]. How SUMO and ubiquitin differentially affect the ability of the PBD to interact with different substrates, regulators or the KD of Plk1 has not been investigated.

## 4. Nuclear Polo promotes mitotic entry

Once in the nucleus, what does Polo do? In *Drosophila*, it was recently shown that Polo needs to enter the nucleus to activate Cdc25 (String) and trigger its relocalization from the nucleus to the cytoplasm, where Cdc25 activates Cdk1 [38]. String is the only form of Cdc25 expressed in *Drosophila* somatic cells. In vertebrates, three genes encode Cdc25 paralogues (Cdc25A, B and C), and they have all been shown to respond to Plk1 activity [46]. Pioneering work in *Xenopus* extracts established that Plx1 phosphorylates and activates Cdc25C, and that this event is required for activation of Cyclin B–Cdk1 [15,16]. Recent work in human cells revealed that Plk1 phosphorylates Cdc25C at multiple sites in its N-terminal region during mitotic entry. Overexpression of a phosphomimetic Cdc25C variant promotes mitotic entry even when Plk1 activity is inhibited, indicating that activation of Cdc25C by Plk1 is sufficient for mitotic entry [24]. It is not clear to what extent Plk1 can also promote mitotic entry by targeting Cdc25A or Cdc25B. Intriguingly, unlike *Drosophila* Cdc25 which is excluded from nucleus following its activation by Polo, human Cdc25C and Cdc25B have been reported to be imported into the nucleus upon phosphorylation by Plk1 [47,48]. That the spatio-temporal regulation of mitotic entry would unfold in completely opposite manners in flies versus humans would be surprising. The spatio-temporal dynamics of the multiple forms of Cdc25 enzymes in humans have not been completely explored, but it is known that Cdc25A and Cdc25B are predominantly nuclear in interphase [49–53]. In *C. elegans*, CDC-25.1, which is essential for embryogenesis, is enriched in the nucleus in early embryos, where it is activated by PLK-1 during the early embryonic divisions [54]. Thus, the activation of Cdc25 in the nucleus in prophase may be an evolutionarily conserved role of Polo kinase during mitotic entry.

In the nucleus, Polo could also contribute to mitotic entry by additional mechanisms. Wee1 is a Cdk1-inhibitory kinase that resides predominantly in the nucleus [17]. In human cells, Plk1 antagonizes Wee1 by phosphorylating it to induce its ubiquitination and subsequent degradation by the proteasome [55]. Plk1 also phosphorylates cohesins to promote their removal from chromosome arms in prophase [56]. In addition, Plk1 promotes chromosome condensation by p­­hosphorylating condensins [57,58]. Moreover, in multiple species, Polo localizes to the centromere regions starting in prophase, where it could promote the maturation of KTs before NEB to allow their correct attachment to microtubules after NEB [35,37,59]. The importance of bringing Polo activity in the nucleus before NEB for these other functions, and to what extent each event is required for the normal completion of mitosis, has not been dissected in detail (figure 1, point 7).

Polo has been linked to Greatwall (Gwl), a kinase that promotes mitotic entry by antagonizing protein phosphatase 2A in complex with its B55 regulatory subunit (PP2A-B55). As the cell enters mitosis, Gwl becomes active and phosphorylates endosulfine proteins (ENSA and Arpp19 in humans). Once phosphorylated, endosulfines selectively inhibit PP2A-B55, a phosphatase that efficiently targets several mitotic phosphosites [60]. In *Drosophila* and in human cells, Gwl is nuclear in interphase, but in prophase it re-localizes to the cytoplasm where PP2A-B55 is enriched. This localization dynamics is important for Gwl function [61,62]. In *Drosophila*, phosphorylation of Gwl by both Polo and CDK activities promotes Gwl relocalization to the cytoplasm just before NEB [61]. However, this regulation of Gwl was shown to depend on CDK but not Plk1 activity in human cells [62]. Nevertheless, results suggest that Plx1 contributes to activate Gwl during DNA damage checkpoint recovery in *Xenopus* [63]. While these elements point at a collaboration between Polo and Gwl in promoting mitotic entry, genetic interactions in *Drosophila* suggest that Polo can function against Gwl and with PP2A-B55 in regulating mitosis [64,65]. The precise mechanisms linking Polo and the Gwl–PP2A module remain to be elucidated.

In addition to entering the nucleus in prophase, Polo is recruited to the nuclear envelope (NE). In worms and flies, this localization of Polo is striking [37,66]. Work in *C. elegans* revealed that PLK-1 is required for NEB in both meiosis and mitosis [67,68]. Recent studies showed that PLK-1/Plk1 is recruited specifically to the nuclear pore complexes (NPCs) in *C. elegans* and in human cells (figure 1, point 8) [37,69]. In both systems, the PBD of PLK-1/Plk1 interacts with nucleoporins primed for by phosphorylation at CDK sites (figure 1, point 9). In *C. elegans*, PLK-1 also phosphorylates nucleoporins to create additional PBD binding sites [37]. PLK-1 recruitment to the NE does not require its nuclear import because PLK-1 still localizes to the NE upon inactivation of importins. However, the disruption of the reciprocal inhibitory interaction between the KD and the PBD induced by Polo phosphorylation on the activation loop is likely the trigger for the PBD to interact with phosphorylated nucleoporins. In human cells, NPC-localized Plk1 promotes the disintegration of NPCs, at least in part, by phosphorylating Nup98 and Nup53, thereby breaching the permeability barrier of the NE (figure 1, point 10) [69,70]. Plk1 may target additional NE components to promote NEB.

In summary, it is now clear that Polo relies on built-in mechanisms by which its activation triggers changes in subcellular localization that are required for Polo to coordinate multiple events in space and time as the cell enters mitosis.

## 5. The upstream trigger: Cyclin A–Cdk1 primes Bora for Polo activation

Now that we understand that Polo kinase activation by phosphorylation also induces its localization to the nucleus, where Polo promotes mitotic entry, it becomes even more crucial to understand the mechanism leading to Polo activation. In human cells, Plk1 phosphorylation on its activation loop requires Bora, which was originally discovered as a cofactor of Aurora A in *Drosophila* in a genetic screen for regulators of asymmetric cell division [71]. Bora accumulates at the G2/M transition in human cells and promotes Plk1 phosphorylation by Aurora A [31,32]. Importantly, this function of Bora in Polo activation is greatly stimulated by its phosphorylation by Cyclin–CDK complexes in both human cells and *C. elegans* [33,34,72,73]. Cyclin–CDK enzymes phosphorylate Bora at multiple sites but phosphorylation of three evolutionarily conserved residues located in the N-terminal, most conserved part of the protein is critical for Bora function in Polo activation, both in *C. elegans* embryos and in human cells [33,72,73]. Thus, Cyclin–CDK enzymes not only orient Plk1 activity by phosphorylating Plk1 substrates to prime their interactions with the PBD, they also directly stimulate Plk1 activation by Aurora A during mitotic entry [34]. In budding yeast, Cdc28/Cdk1 directly activates Cdc5/Polo by phosphorylating its activation loop [74]. Thus, Polo activation by a CDK is conserved from yeast to humans, although Bora and Aurora A act as intermediates in higher eukaryotes.

Which is the physiological Cyclin–CDK complex phosphorylating Bora? Bora contains a conserved cyclin-binding site (Cy) and both Cyclin B–Cdk1 and Cyclin A2–Cdk2 can phosphorylate Bora *in vitro* [33,75]. In human cells, Plk1 is activated before Cyclin B–Cdk1, so even if Cyclin B–Cdk1 can phosphorylate Bora to sustain Plk1 activity during mitosis [76], it is unlikely to be the Cyclin–CDK complex that activates Plk1 in G2. Cyclin A has long been implicated in mitotic entry, but its critical targets in this process have remained unknown [77,78]. Cyclin A associates with Cdk2 in S-phase and then with Cdk1 during G2 before being degraded in prometaphase [79,80]. By an unknown mechanism, Cyclin A partially re-localizes from the nucleus in S-phase to the cytoplasm in G2; moreover, Cyclin A associates with Plk1 and Bora [24,75]. These observations strongly suggested that Cyclin A–Cdk1 phosphorylates Bora in G2 to trigger Plk1 phosphorylation by Aurora A, and in turn mitotic entry (figure 1, point 2).

Recent work in *Xenopus* extracts confirmed this hypothesis and revealed that Cyclin A–Cdk1 phosphorylates Bora to trigger Plk1 activation and mitotic entry [81]. In this system, immunodepletion of either Plk1 or Bora totally prevents mitotic entry [81,82]. Strikingly, mitotic entry can be rescued in Bora-immunodepleted extracts by adding back the N-terminal domain of human or *Xenopus* Bora produced in *Escherichia coli* but not mutated versions of Bora defective in Cyclin binding (mutated on the Cy motif) or lacking the three evolutionarily conserved CDK-dependent SP phosphorylation sites. However, exogenous Bora failed to rescue mitotic entry in extracts also depleted of Cyclin A. By contrast, Bora thiophosphorylated *in vitro*, and thus resistant to endogenous phosphatases, readily supported mitotic entry in extracts depleted of Bora and Cyclin A. These observations unequivocally demonstrate that Bora is the necessary and sufficient Cyclin A target required for Plx1 activation and mitotic entry. Once Cyclin B–Cdk1 is activated in mitosis, it likely sustains Polo activation by phosphorylating Bora as Cyclin B co-immunoprecipitates with Bora in mitotic cells [33]. Knowledge of the precise mechanism by which Bora (and its phosphorylation by Cyclin A–Cdk1) helps activate Plk1 (Polo) is still lacking and will likely require structural biology approaches.

In addition to Bora, the multi-functional adaptor protein WAC contributes to Plk1 activation by Aurora A and to timely mitotic entry in human cells [83]. In contrast with Bora, which is predominantly cytoplasmic, WAC is a nuclear protein. WAC interacts with the PBD of Plk1 in a phospho-dependent manner. Cyclin–CDK enzymes phosphorylate WAC at multiple Polo-docking sites, which in turn promotes WAC binding to the PBD of Plk1. As WAC also binds Aurora A, it can bring together Aurora A and Plk1. In the future, it will be important to determine whether WAC only acts as a platform that bridges Aurora A and Plk1 or whether it has a more active role in promoting Aurora A activation. Overall, these observations suggest the existence of two complementary mechanisms for Plk1 activation by Aurora A: one cytoplasmic that depends on Bora and the other one nuclear that relies on WAC (figure 1, point 6). Both pathways are primed by mitotic Cyclin–CDK enzymes.

## 6. Keeping Polo in check: the role of protein phosphatases

During mitosis in human cells, protein phosphatase 1 (PP1) dephosphorylates Thr210 of Plk1 and thereby antagonizes its activity [84]. This event requires Mypt1, a regulatory subunit of PP1, which is phosphorylated at a CDK site to prime its interaction with Plk1. Recent work showed that Cyclin A–Cdk1 phosphorylates Mypt1 in prometaphase to facilitate the downregulation of Plk1 activity at kinetochores [84,85]. Interestingly, Plk1 can also promote its own inactivation by PP1-Mypt1 in the nucleus, before NEB [86]. This occurs by the phosphorylation of the Golgi-associated protein Optineurin by Plk1, which induces the nuclear import of Optineurin in complex with Mypt1. Disrupting this regulation leads to mitotic defects. The precise role of this proposed negative feedback in Plk1 regulation is still unclear. As Plk1 needs to be active in the nucleus to promote mitotic entry, this inhibition could help deter premature mitotic entry and ensure a timely switch-like activation of Plk1 in the nucleus at the G2/M transition by the mechanisms discussed above. Cyclin A, which promotes S-phase and is primarily nuclear during interphase [87], would be ideally poised to keep Plk1 in check until DNA replication is completed by phosphopriming Mypt1. In addition to PP1-Mypt1, other phosphatases can contribute to dampen Plk1 activity. For example, PP2A-B55α dephosphorylates Plx1 in response to the DNA damage response in *Xenopus* extracts [88]. As PP2A-B55*α* is mainly in the cytoplasm [89], it may interfere with the initial activation of Plk1 that takes place in that compartment. Further work will be required to fully understand how phosphatasaes contribute to the spatio-temporal regulation of Polo kinase.

## 7. Conclusion and perspective

Although still incomplete, the new knowledge of the complex mechanisms enforcing spatio-temporal control of Polo kinase during mitotic entry constitutes a major leap forward in our understanding of cell division at the molecular level. Much work remains to be done to comprehend the regulation and precise functions of Polo kinase in late phases of cell division, including cytokinesis. Understanding the mechanisms regulating Polo opens possibilities to selectively disrupt specific regulatory modes or downstream functions of Polo in order to dissect their importance experimentally. This can be achieved by mutations at specific sites in Polo, inactivation of upstream regulatory enzymes or even the development of small molecule modulators as tool compounds. Several efforts have been deployed to develop PBD inhibitors that could be used to assess the importance of PBD interactions with targets for specific functions of Polo [90]. The development of chemical inhibitors of the NLS of Plk1 is also being attempted [91]. Although these early endeavours have so far failed to produce highly selective, cell-permeable compounds, the future could bring surprising developments. The sustained interest in Plk1 as a target for potential anti-cancer drugs may further enhance this research direction [90].

## Data accessibility

This article has no additional data.
